# Multifunctional Fischer Aminocarbene Complexes as Hole or Electron Transporting Layers in Organic Solar Cells

**DOI:** 10.3390/molecules23040751

**Published:** 2018-03-24

**Authors:** Pablo Vidal-García, María Elena Sánchez-Vergara, Ricardo Corona-Sánchez, Omar Jiménez-Sandoval, Efraín Gutiérrez-Rivas Mercado, Rubén A. Toscano, Cecilio Álvarez-Toledano

**Affiliations:** 1 Universidad Anáhuac México, Campus Norte, Engineering Department, Avenida Universidad Anáhuac 46, Col. Lomas Anáhuac, Huixquilucan 52786, Estado de México, Mexico; pablo94vg@gmail.com (P.V.-G.); efragrm@gmail.com (E.G.R.M.); 2 Instituto de Química, Universidad Nacional Autónoma de México, Circuito Exterior, Ciudad Universitaria, Ciudad de México 04510, Mexico; ricardo_corsan@yahoo.com.mx (R.C.-S.); toscano@unam.mx (R.A.T.); 3 Centro de Investigación y de Estudios Avanzados del Instituto Politécnico Nacional, Unidad Querétaro, Libramiento Norponiente 2000, Fracc. Real de Juriquilla, Querétaro 76230, Querétaro, Mexico; ojimenez@cinvestav.mx

**Keywords:** Fischer aminocarbene complexes, pyridine derivatives, optoelectronic properties, hole transporting layers, electron transporting layers

## Abstract

A new series of Fischer carbenes have been synthetized and examined as hole-transporting or electron-transporting layers (HTLs or ETLs) in the fabrication of organic solar cells (OSCs). The synthesis of three Fischer aminocarbene complexes with the general formula [Cr(CO)_5_{C(NHCH_2_)Ar}] (Ar = 2-pyridyl (**3a**), 3-pyridyl (**3b**) and 4-pyridyl (**3c**)) is reported. The molecular structure of complex **3b** has been confirmed by X-ray analysis. In order to study the possible applications of the three Fischer aminocarbenes in OSCs, thin films of these complexes were prepared using a vacuum deposition process. These organometallic films were chemically and morphologically characterized by IR spectroscopy, SEM, AFM and XRD. According to the IR and Tauc analysis, the vacuum deposition process generates thin films free of impurities with an activation energy of 4.0, 2.7 and 2.1 eV for **3a**, **3b** y **3c,** respectively. The UV-vis spectra of the amorphous aminocarbene films show that they are practically transparent to the visible radiation of the electromagnetic spectrum. This is due to the fact that their absorption is located mainly in the ultraviolet range. Two OSCs with bulk-heterojunction configuration were manufactured in order to prove the use of the aminocarbenes as ETL o HTL. The aminocarbene [Cr(CO)_5_{C(NHCH_2_) 4-pyridyl}] (**3c**) proved to be suitable as ETL with a fill factor (FF) of 0.23 and a short circuit current density (*J_SC_*) of 1.037 mA/cm^2^.

## 1. Introduction

In commercial optoelectronics there are several types of *p*-*n* single-junction two-terminal devices such as Schottky diodes, varactor diodes, light emitting diodes (LEDs), and solar cells. In a certain way LEDs and solar cells can be considered to be devices that work in a reverse mode, i.e., while in the LED light is obtained from electric energy and the charge carriers travel from the electrodes to the emissive material, where light is produced by the radiative recombination of an exciton electrically generated, the efficiency of the solar cell results from the relationship between the number of charge carriers generated and the number of incident light photons. In recent years, the interest in the development of solar cells as an alternative source of energy has increased, however, further research and development are required in order to maximize the conversion efficiency of solar energy into electrical energy. More specifically, organic photovoltaics (OPV) is an area of great promise, as it is rapidly maturing through new research in organic semiconductors [1]. This type of semiconductors exhibits an energy gap between the Highest Occupied Molecular Orbital (HOMO) and Lowest Unoccupied Molecular Orbital (LUMO) that lies within the range of inorganic semiconductors (1–3 eV). The most important distinctive characteristic respect to inorganic semiconductors, is the non-covalent nature of the intermolecular interactions governing the ordering in the solid state. The weakness of these interactions hinders the formation of an ordered structure that favors the optimal overlap of orbitals belonging to molecules located at a very close range. Consequently, except in exceptional cases, an energy band structure is not favored, and the energetic levels will be defined by levels located in each molecule [2]. Even so, within the context of semiconductors, an energetic parallelism between valence and conduction bands and molecular orbitals can be established. The HOMO will relate to the superior limit of the valence band and will always be separated from the LUMO by an energetic spacing, which corresponds to the inferior limit of the conduction band. Due to the absence of bands, the movement of electrons occurs by a charge transfer between levels located in the molecules of the organic semiconductor, which although it hinders the electric current (*I*) in comparison to inorganic semiconductors, generates enough current density (*J*) to make them suitable for manufacturing optoelectronic devices such as organic solar cells (OSCs). It is important to mention that while high power conversion efficiencies of even 13.2% have been reached [3,4], small exciton diffusion lengths, narrow absorption bands, low charge carrier mobility [5] and poor load transport still affect the operation of OSCs. The basic structure of an OSC includes an anode, an n-type semiconductor, a p-type semiconductor and a cathode. The transport of load carriers from the electrodes to the semiconductor active layers can be improved by using additional layers. Incorporating interfacial layers between the electrodes and the active layers benefits the contact between them, having enhancing effects in the charge extraction and recollection processes [2]. In addition, they can determine the relative polarity of the device, since one same electrode can act as anode or cathode, depending on the nature of the interfacial material used [2]. Two types of interfacial processes can be achieved: the improvement of the transport of either holes or electrons, by using hole- or electron-transporting layers (HTLs or ETLs), respectively. This involves the use of organic semiconductors with optoelectronic properties which selectively stimulate the conduction of one type of carrier [6]. Different materials have been used as organic semiconductors, such as metallophthalocyanines (MPcs), tetrathiafulvalene (TTF) [7], perovskites [8], fullerene [9] and tetracyanoquinodimethane (TCNQ) [10]. However, only a few materials such as carbazoles [6], copper phthalocyanine (CuPc) [11], pentacene [12], carbon nanotubes [13] and poly(3,4-ethylenedioxythiophene) (PEDOT) have been studied as HTLs, [14]; or as ETLs (e.g., bathocuproine (BCP) [15,16], naphthalic anhydride and bathophenanthroline (BPhen) [2]). Aditionally polymer solar cells are prepared with the active polymer donor–fullerene acceptor blend sandwiched between a HTL and an ETL [4].

On the other hand, since their synthesis by Fischer and Maasböl in 1964 [17], group 6 Fischer metal-carbene complexes have been exceptionally useful as synthons in organic and organometallic syntheses [18,19,20,21]. Fischer’s carbene ferrocenyl complexes have been previously studied by important groups of researchers as Barluenga et al. [21], Sierra et al. [22,23,24,25,26,27,28] and Landman et al. [29,30]. Some authors of this work, have studied their optical and electric properties for their use as semiconductors [31], however, their application as components of optoelectronic devices has been little analyzed and even less their use as HTLs or ETLs for OSCs. HTLs are incorporated into OSCs in order to allow the transfer of the positive charge carriers injected by the anode of the device or as an electron blocking layer, while the ETLs are responsible for the transport of the electrons injected by the cathode of the device and also act as blockers of holes [2]. Due to the above, it would be expected that Fischer metal-carbenes behave as ETLs since they form stable radical anions [28] and could transport electrons. Given the interest that the development of novel organic semiconductors with application in the manufacture of OSCs presents, this work mainly focuses on the synthesis and characterization of Fischer aminocarbene complexes, the evaluation of their optical and electrical properties and their use in the manufacture of OSC type optoelectronic devices. In particular, the different structures of aminocarbene complexes as hole- or electron-transport layers for OSCs are analyzed. 

## 2. Results and Discussion

### 2.1. Synthesis of the Organometallic Precursors

**Scheme 1:**
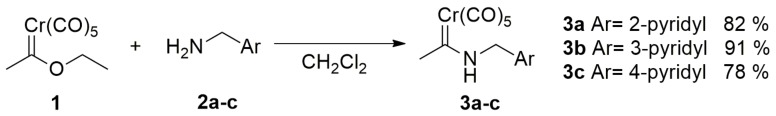
Synthesis of aminocarbene complexes.

The pyridyl(aminocarbene)chromium(0) complexes **3a**–**c** were synthesized in good yields using the classical aminolysis method reported by Fischer (Scheme 1) [17].

The formation of complexes **3a**–**c** was readily accomplished by adding an excess of the required (aminomethyl)pyridine **2a**–**c** to a dichloromethane solution of [(ethoxy)(methyl)methylidene]-pentacarbonyl chromium (0) **1** at room temperature. The product precipitated out of the solution as a solid, allowing for effortless purification. Fischer aminocarbenes **3a-c** are air-stable solids at room temperature and their structure was confirmed by solution NMR spectroscopy. According to its physical data, pyridylamino carbene complex **3a** exists as a mixture of *E*- and *Z*-isomers with respect to the partial C-N double bond, in a 2:1 approximate ratio (determined by ^1^H-NMR integration), in accordance with related complexes previously reported [28]. For complexes **3b** and **3c**, a single isomer was detected. The ^1^H-NMR spectra of complexes **3a**–**c** show a broad signal around 11.0 ppm attributable to the N-H proton, a set of signals from the four different protons of the pyridine nucleus, a doublet signal around 4.98 ppm from the methylene group and the signal of the methyl group associated with the carbene carbon at 2.8 ppm. The ^13^C-NMR spectra confirmed the formation of the aminocarbene complexes by the signal of the carbene carbon appearing around 278 ppm and the metal carbonyl signals (Cr-CO) at 223 and 218 ppm. The infrared spectra display the three M-CO bands typical of a metal pentacarbonyl system at 2050, 1977 and 1903 cm^−1^ (Cr-CO), as well as a weak band at ca. 3170 cm^−1^ assigned to vibrations of the N-H group. 

**Figure 1:**
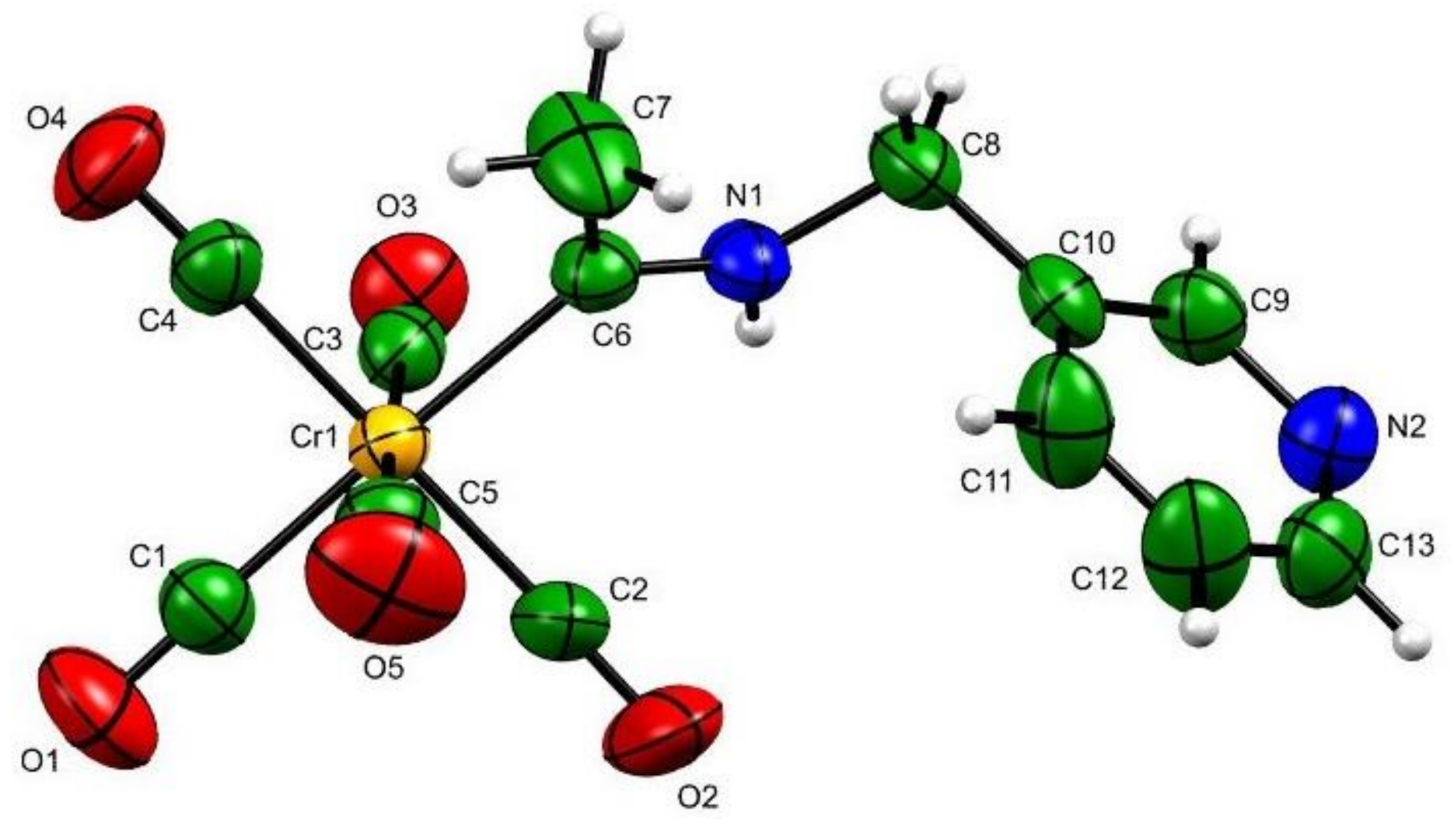
ORTEP view of complex **3b**. Thermal ellipsoids at 30% probability level.

Additionally, the peaks observed by HRMS (ESI-TOF) were in agreement with the presence of five carbonyl groups and confirmed the expected masses in accordance with their molecular formule; it was found that the pyridine fragment is not coordinated to the metal, unlike what has been reported for other related aminocarbenes [32]. Besides, the structural arrangement for **3b** was unequivocally established by single-crystal X-ray diffraction analysis (Figure 1 and Table 1). 

Since aminocarbenes **3a**–**c** differ in the position of the pyridyl group, we envisaged that the optical and electrical properties of thin films prepared with these complexes could vary considerably depending on the substitution position of the pyridine ring.

### 2.2. Preparation and Characterization of the Aminocarbene Thin Films

The manufacture of the multilayer structure OSCs proposed in this work, was carried out by using the following components: thin films of the aminocarbene complexes prepared, to function as electrodes, the active layer, and the selective hole- or electron-transport layers. Thin films favor the charge transport; they are compact, lightweight, and since a dimension is neglected, they are easier to characterize physically. 

**Figure 2:**
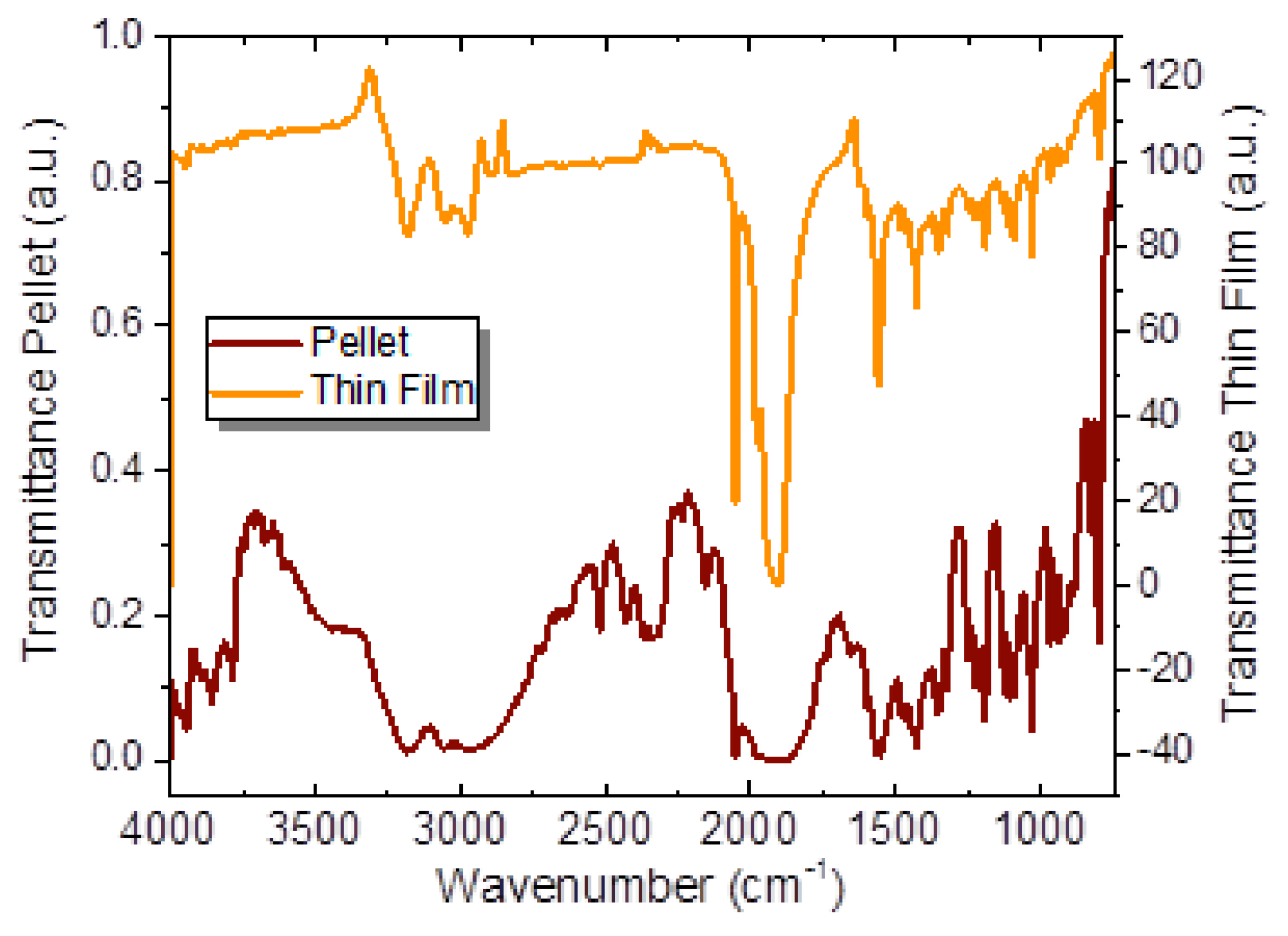
IR spectrum of aminocarbene **3b** (3-pyridyl derivative) as KBr pellet and thin film.

In order to analyze the possibility of using Fischer carbenes as a functional element of multilayer OSCs, they were deposited and later characterized as thin films. The FT-IR spectroscopy analysis performed on the thin films deposited on monocrystalline silicon wafers provided information about the chemical structure and purity of the aminocarbene complexes. The spectra of the films show a band around 3170 cm^−1^, assigned to the N-H group, as well as bands about 1902 and 2050 cm^−1^, corresponding to Cr-C=O vibrations; the small band at 1975 cm^−1^ was only evident in the spectrum of compound **3b** (Figure 2), while in the other two cases it is assumed to be masked by the wide band at 1902 cm^−1^. The shifts between the position of the signals of the films, compared to those of the compounds as KBr pellets, observed in Figure 2 for aminocarbene **3b** (similar spectra were obtained for complexes **3a** and **3c**), are attributed to residual efforts in the films, as a result of the thermal gradient to which the Fischer carbenes are subjected during the evaporation process. The infrared spectra also reveal that the films obtained do not contain traces of reaction products, so the high vacuum conditions used, generate films free of impurities that could affect the molecular stacking and create regions with energetic levels different from HOMO and LUMO. 

**Figure 3:**
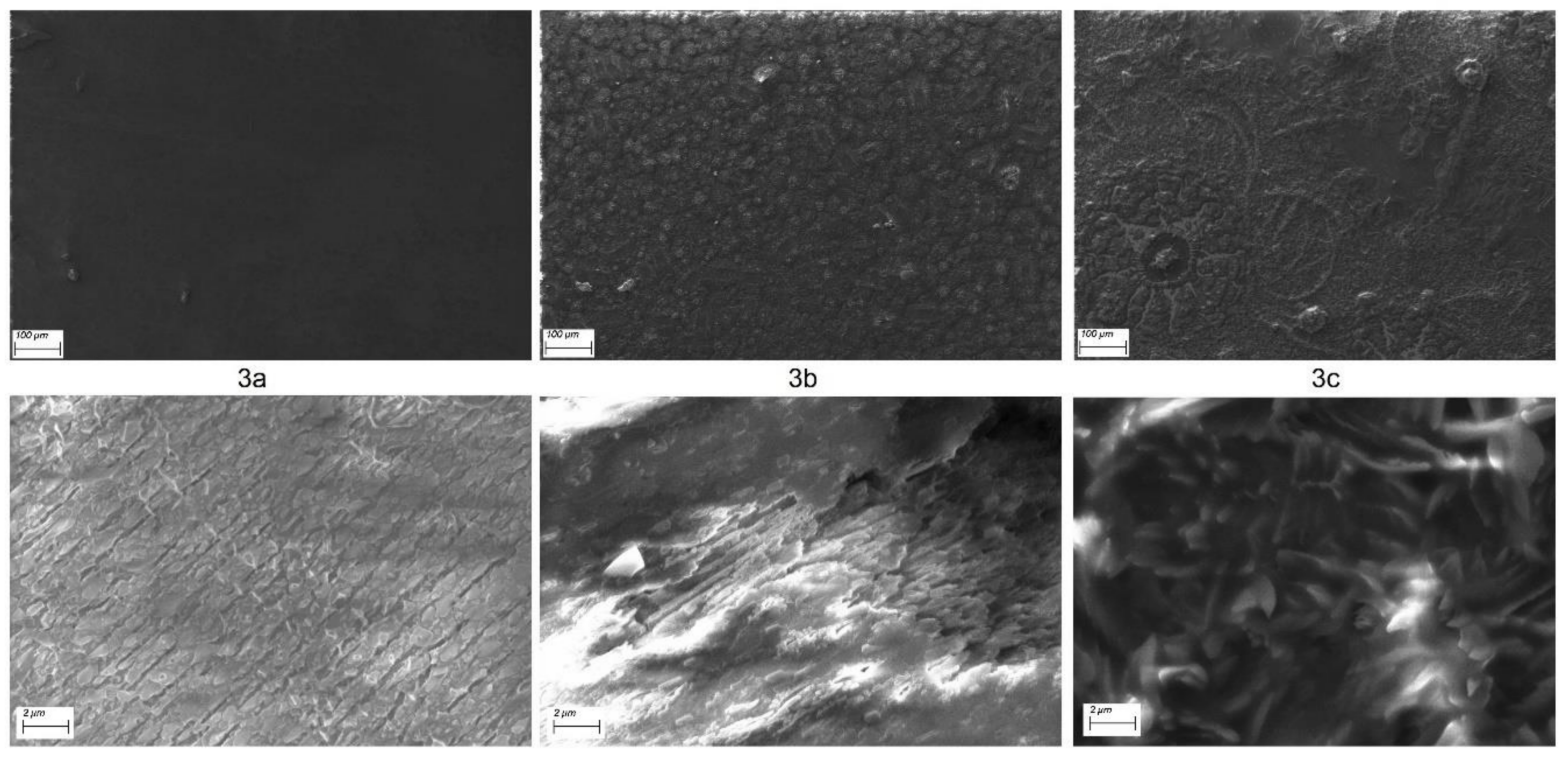
SEM images of the aminocarbene complexes thin films (**3a** = 2-pyridyl; **3b** = 3-pyridyl; **3c** = 4-pyridyl derivative) at 100× and 5000×.

Another important aspect to consider is the morphology of the thin films; if efficient charge transport properties are sought after, homogeneous films are required. Figure 3 shows the SEM images of the three films deposited over Corning glass substrates at 100× and at 5000× magnifications. The appearance of the film of Fischer aminocarbene **3a** at 100× is homogeneous with some larger structures on the surface; in the 5000× image of the same film it is clearly observed a preferential direction growth. The image of the carbene **3b** film at 100× shows a surface constituted by particles of different size and predominantly rounded shape; in addition, the corresponding image at 5000× exhibits a directional growth, similar to film **3a**. Finally, for the case of aminocarbene **3c**, the heterogeneity observed at 100× is very clear: there are zones with rounded as well as elongated structures of different sizes. The image for the same film obtained at 5000× reveals a fibrous morphology with growth in several directions. Since the preparation conditions were the same for all the thin films (arrangement of the substrates in the evaporation chamber, temperature, pressure and deposition velocity), it is apparently the chemical structure and, more specifically, the position of the pyridyl group in the Fischer carbene molecule, responsible for the morphology of the films. 

The nucleation and directional growth during the preparation of the films, may be indicative that the carbenes [Cr(CO)_5_{C(NHCH_2_)Ar}] exhibit some anisotropy in their optoelectronic properties [33,34,35].

**Figure 4:**
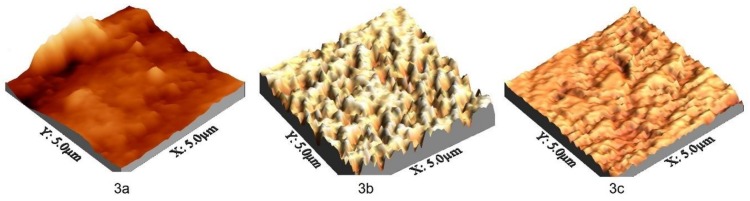
AFM images of the aminocarbenes films (**3a** = 2-pyridyl; **3b** = 3-pyridyl; **3c** = 4-pyridyl derivative).

In order to complement the information regarding the morphology of the films, AFM studies were performed. Figure 4 shows the images obtained. An irregular morphology can be observed for all the films, constituted by multiple peaks and valleys. Film **3a** exhibits an irregular texture with rounded particles of different size and scattered on the surface. Film **3b** shows smaller aggregates forming a denser film; although some clear spots can still be seen, the film exhibits a nearly complete coverage of the substrate. On the other hand, thin films from sample **3c** exhibit very small aggregates of similar appearance and a total surface coverage can be observed. From the AFM images, it is clear that the chemical structure of the complex defines the aggregation state on the substrates. 

The topography obtained showed root mean square (rms) roughness values of 527 nm for film **3a**, 166 nm for film **3b**, and 481 nm were found for film **3c**. Such differences in roughness could be correlated with the position of the pyridyl group in the carbene and the form of nucleation and growth over the substrate. It is important to mention that for an efficient charge transport to occur, low roughness films are preferred, such as that obtained with complex **3a**. A low roughness protects to a greater extent the active layer of the OSCs from the chemical and physical interaction with the electrode materials, oxygen and water from the atmosphere. In addition, for electrodes whose surfaces have a certain roughness, such as the ITO (Indium tin oxide) in the present study, low roughness layers of HTLs or ETLs help to soften their surface, providing a more appropriate morphology for a good contact with adjacent layers. The Fischer aminocarbene films in this work have a somewhat irregular morphology, the complexes have been deposited on the entire surface of the substrate, practically free of holes or external impurities. The potential of these films to be used as HTLs or ETLs is mainly determined by their optical and electrical properties.

### 2.3. Optical Properties of the Fischer Aminocarbene Films

**Figure 5:**
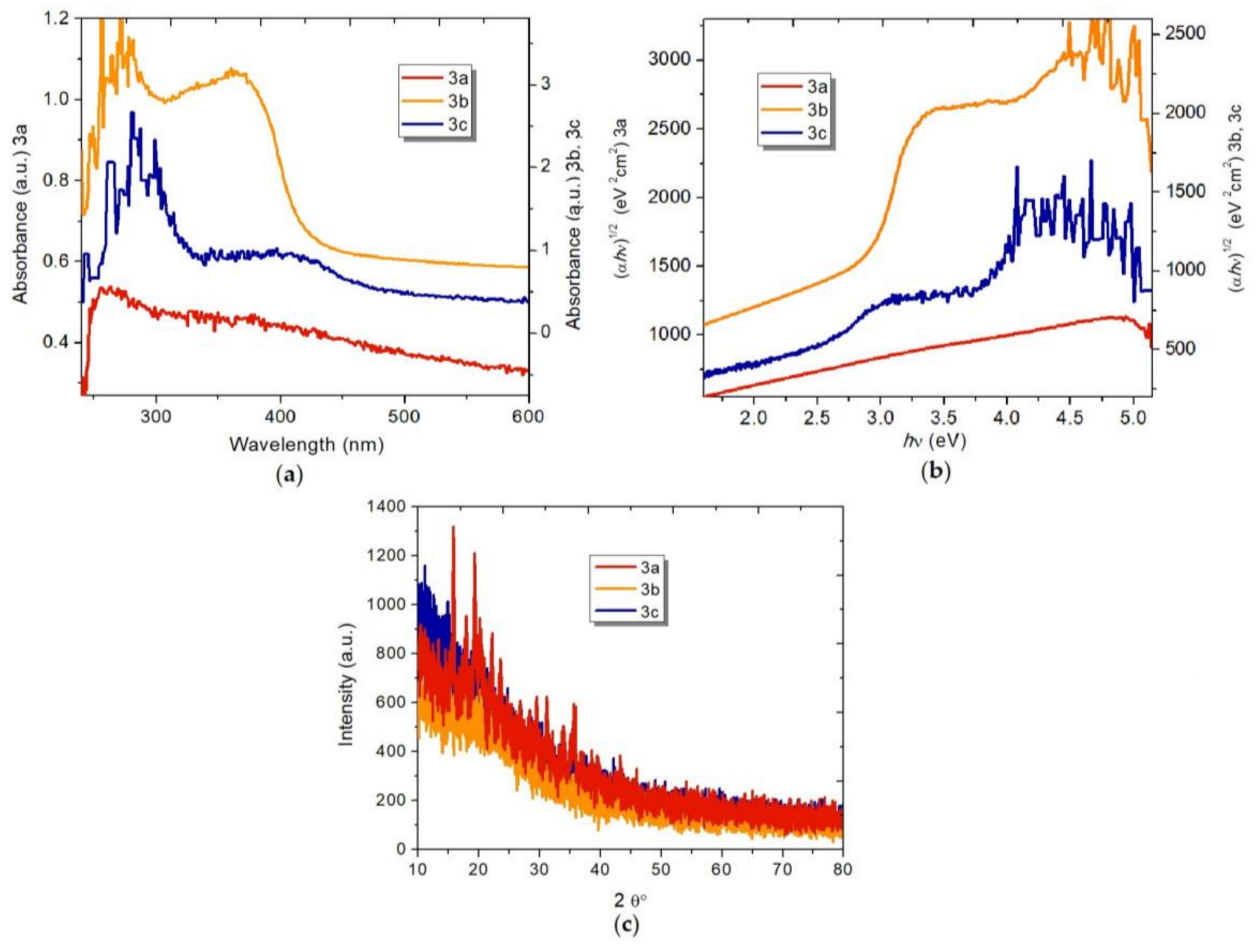
(**a**) Absorption spectra of the films in the 240–600 nm range; (**b**) Absorption curves showing the transitions that correspond to indirect band gaps in the thin films; and (**c**) XRD patterns of the films.

From the viewpoint of the application of carbenes as components of OSCs, where the absorption-emission of radiation is the principle on which their operation is based, the study of their spectroscopic properties is of great importance. The different electronic transits taking place between the fundamental and excited states, as a consequence of the absorption of radiation, are registered by UV-vis spectroscopy. Clear differences can be observed in the absorption spectra of the films, Figure 5a: while complex **3a** shows a maximum around 260 nm, thin films of compounds **3b** and **3c** show two peaks; the first one corresponds to a strong absorption between 270 and 280 nm, and the second one, to a weak absorption between 360 and 390 nm. These peaks with maximum absorption in the ultraviolet region of the spectrum, may correspond to the π-π* transitions among the different vibrational levels in the first singlet excited state and the ground state. In accordance with Kayunkid et al. [36], the above cannot be attributed to temperature changes during the formation of the films, since all the substrates are kept at room temperature. Hence, the key change inducing factor in the absorption properties of the films should be associated with the pyridyl position in the molecule. On the other hand, a fundamental requirement for Fischer carbenes be used as HTLs or ETLs is that they are transparent to visible radiation, in order to fulfill their optical spacer function without reducing the amount of radiation that can be absorbed. Figure 5a shows that the three films are practically transparent to the visible radiation of the electromagnetic spectrum because their absorption peaks are located mainly in the ultraviolet range [37,38]. Apparently, the amorphous nature (Figure 5c) of the films generates this transparency in the carbenes. Therefore, aminocarbene films could be used in the manufacture of optoelectronic devices that require transparent circuits. 

The energy difference between the HOMO and LUMO, i.e., the optical activation energy or bandgap, is a parameter that provides information to evaluate the semiconductor properties of the Fischer carbenes. The value of the optical activation energy, attributed to the lower energy transition that takes place by a photon absorption, is estimated by UV-vis spectroscopy. In order to calculate the energetic spacing, different methods are applied, the most common for amorphous films being the Tauc method [7,10,39]. It consists in the extrapolation of the linear stretch of the skirt of the lower energy band of the absorption coefficient (α*hv*)^1/2^ for indirect transitions (see Figure 5b), up to the cutoff point with the abscissa axis corresponding to the energy of the photon (*hν*). In amorphous semiconductors there is no conservation of the electronic momentum, since the optical transitions are basically indirect transitions from states in the HOMO to states in the LUMO [39]. Although according to SEM, the growth of the thin films was in preferential directions, no crystalline structure was observed by X-ray diffraction, Figure 5c. This is probably due to two factors: the thermal gradient between the evaporated carbenes and the substrates, as well as the high growth velocity of the nuclei, which does not allow for an ordered structure to be achieved. Although film **3a** has a certain degree of crystallinity, the lack of a complete order does not allow it to be treated as a crystalline semiconductor. The energy values of 4.0, 2.7 and 2.1 eV for **3a**, **3b** and **3c** semiconductor films, respectively, are obtained from the graphs shown in Figure 5b. The three activation energies are within the range of those required for organic semiconductors used in the manufacture of optoelectronic devices [2,10,15]. Nevertheless, in the case of complex **3a**, an activation energy of 4 eV was estimated and this makes it less feasible to be used as HTL or ETL, since its mobility is low and would not favor the transport of the corresponding carriers. The above is complemented with the study of the electrical behavior *J*-*V* of the aminocarbene films.

### 2.4. Electrical Properties of the Fischer Aminocarbene Films

**Figure 6:**
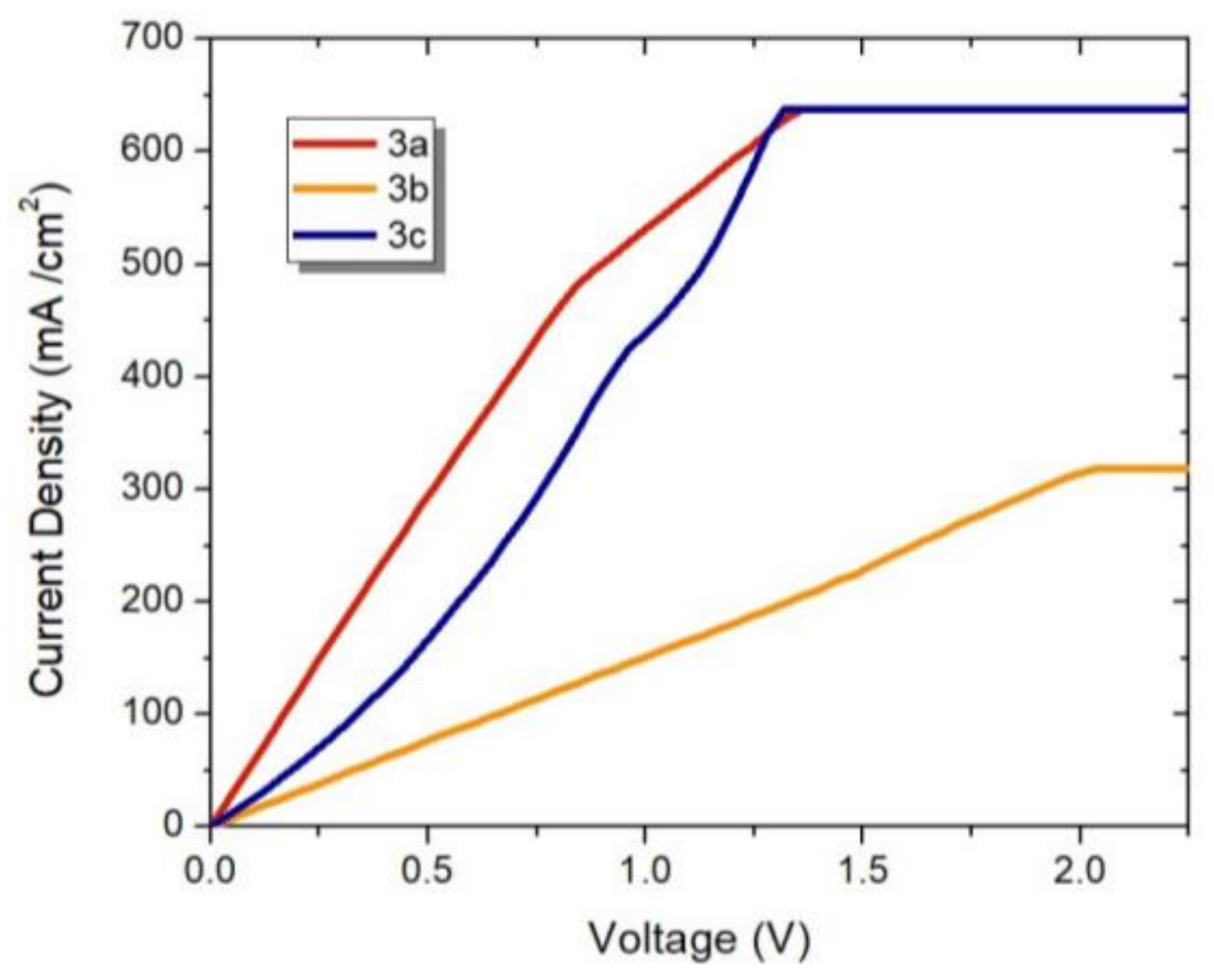
*J*-*V* characteristics of **3a**, **3b** and **3c** thin films under white light illumination, at room temperature (**3a** = 2-pyridyl; **3b** = 3-pyridyl; **3c** = 4-pyridyl derivative).

The three structures containing only Fischer aminocarbenes (glass/ITO/**3a**/Ag, glass/ITO/**3b**/Ag and glass/ITO/**3c**/Ag) were submitted to current-voltage (*I*-*V*) measurements to obtain their current density vs. voltage (*J*-*V*) characteristics, shown in Figure 6. The three are governed under an ohmic law, acting as semiconductors until they reach saturation [2]. Film **3a** decreases its performance at 0.84 V, diminishing its slope. Aminocarbene **3b** has a constant slope, but it is significantly lower, reaching saturation at 2 V, with a current density of 320 mA/cm^2^. Film **3c** shows a better overall behavior, slightly improving its performance at 0.96 V. Both films **3a** and **3c**, reach a maximum current of 630 mA in saturation at 1.3 V. Thin film **3b** is discarded to be used as a selective transport layer because of the small current density it allows. Taking this into consideration, that **3a** and **3c** films saturate at 1.3 V and allow a maximum current density of 630 mA/cm^2^, both could act as interfacial materials. Nevertheless, **3c** is the best candidate to elaborate OSCs, as it saturates at the same voltage than **3a**, but shows a more constant slope. Additionally, film **3a** has an activation energy superior to 4 eV, which makes it inefficient for charge transport, since its energy gap between HOMO and LUMO is high. On the other hand, the preferential direction growth that films **3a** and **3b** show makes them susceptible to present an anisotropy in their optoelectronic properties; this anisotropy generates a preferential direction by which electric charges circulate, however, the charge transport can be null in other directions [33,34,35]. This makes inefficient the function of **3a** and **3b** as HTLs or ETLs, where although there is a process of selective extraction of charge carriers, such process is more related to the mobility of hollows to the anode and electrons to the cathode.

### 2.5. Electrical Properties of the OSCs

**Figure 7:**
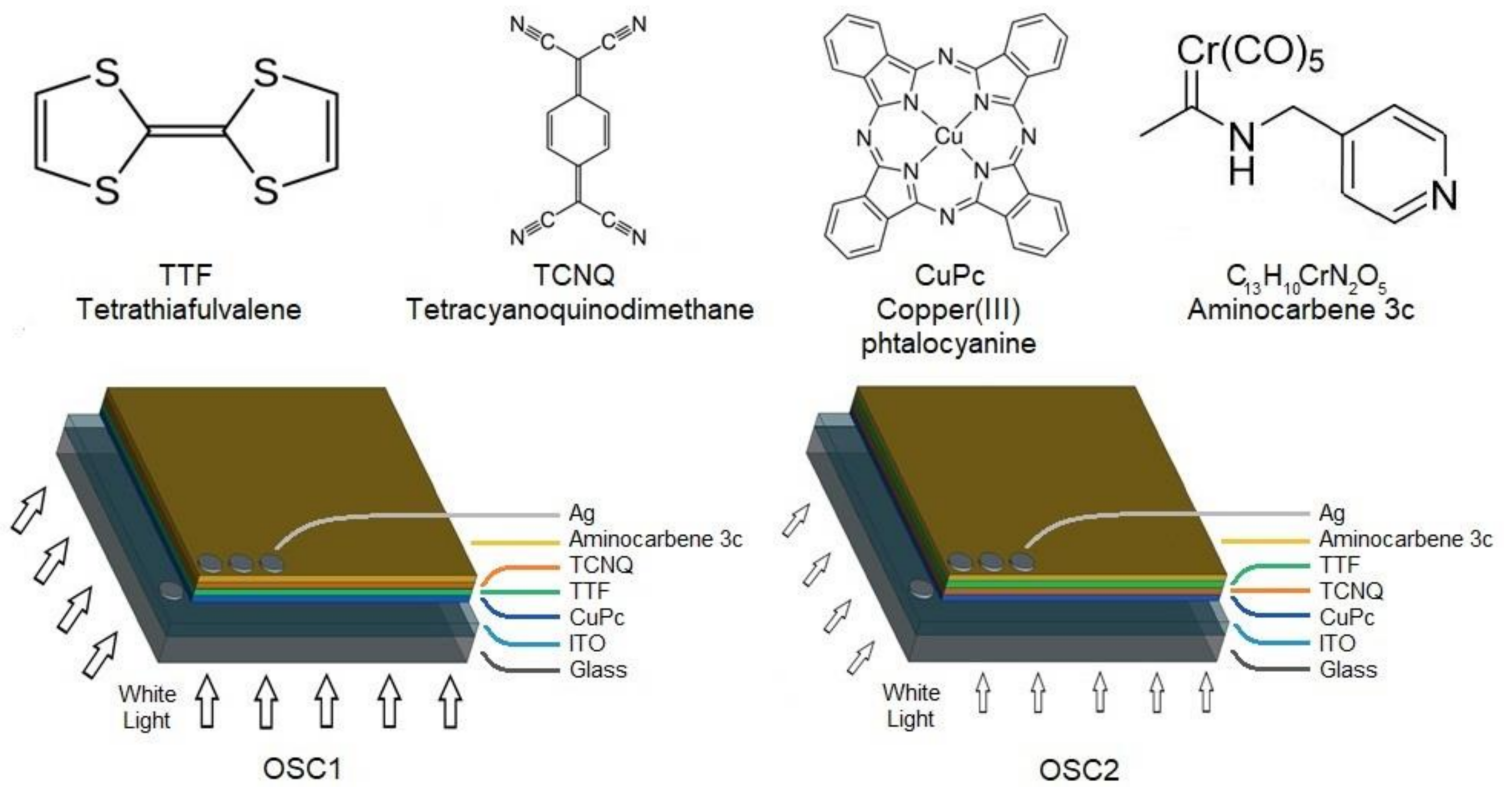
Schematic diagram of the configuration of the two OSCs constructed and the molecular structures of the materials used.

In order to evaluate if the Fischer aminocarbene **3c** can work as an interfacial layer, two OSCs with this material were constructed and analyzed. They were fabricated on glass substrates coated with semi-transparent indium tin oxide, ITO, and an Ag electrode was deposited on the top. Figure 7 shows the configuration of the two bulk-heterojunction photovoltaic devices, OSC1 and OSC2, together with the chemical structure of the semiconductors used. The compounds were: Tetrathiafulvalene (TTF, Sigma-Aldrich, St. Louis, MO, USA), Tetracyanoquinodimethane (TCNQ, Sigma-Aldrich), Copper(II) phthalocyanine (CuPc, Sigma-Aldrich) and aminocarbene **3c** [Cr(CO)_5_{C(NHCH_2_) 4-pyridyl}] (synthetized in the current study). Because of the position of the carbene in the sandwich structures, it is expected to act as an ETL in OSC1 and as an HTL in OSC2.

**Figure 8:**
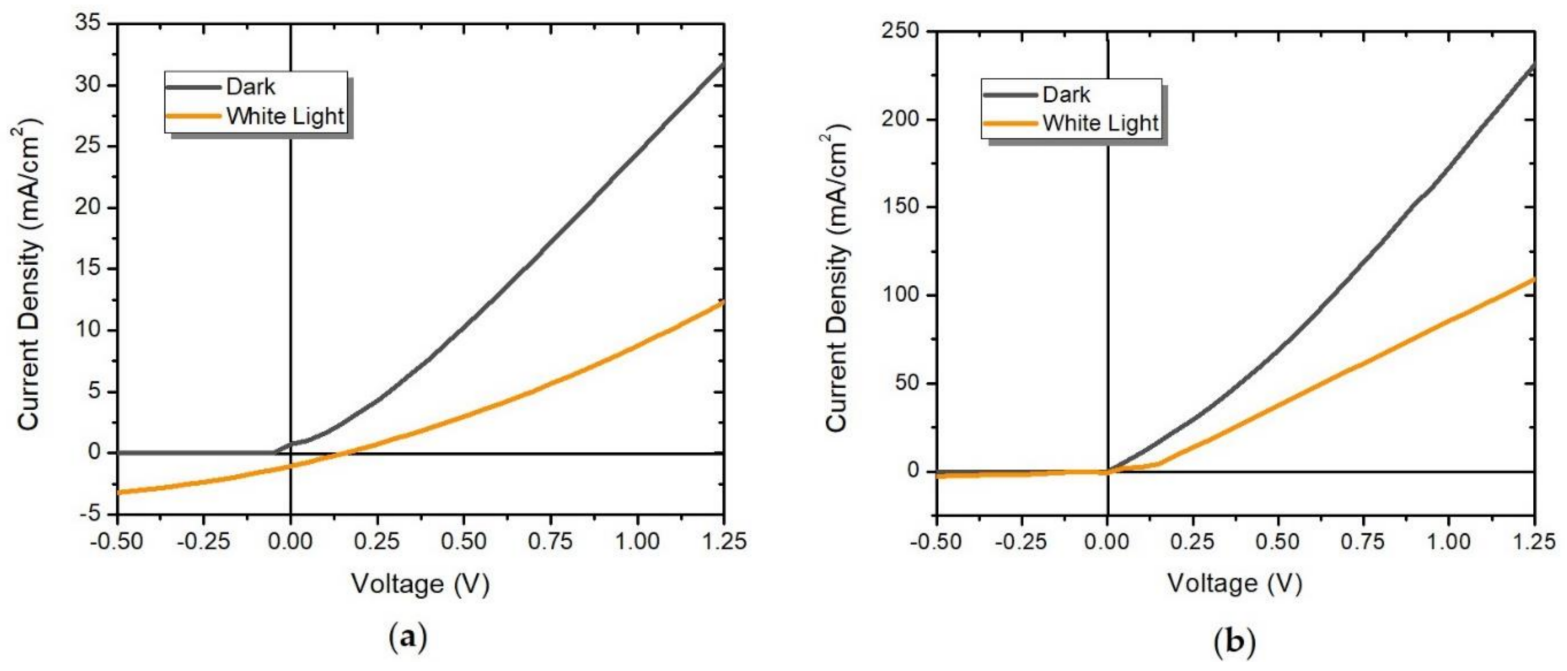
*J*-*V* characteristics of (**a**) OSC1 and (**b**) OSC2.

Commonly, the quality of a solar device is given by the parameter known as fill factor (FF) [40], which indicates how effective the transport and recombination processes are. In our case, the FF will be taken as the reference point to analyze whether the Fischer aminocarbenes act or not as effective cathodic or anodic interfacial layers [2]. FF is determined as the relation between the maximum power and the power obtained when the cell acts as a diode:

(1) $$\mathrm{FF}=\frac{V_{\max}J_{\max}}{V_{OC}J_{SC}}$$

where *V_max_* and *J_max_* are the maximum voltage and current density, respectively, found at the 4th quadrant of the *J*-*V* characteristics [41]. *V_OC_* and *J_SC_* are the open circuit voltage and short circuit current density [41], respectively, which can also be obtained from the *J*-*V* curves. The results shown in Figure 8 and Table 2 were obtained with the cells at room temperature in darkness and under white illumination. Just by observing Figure 8, the behavior of a solar cell can be recognized for OSC1. The variation between the dark and illuminated curves in the 4th quadrant is clear, anticipating a fill factor that would indicate that the device acts as a solar cell. On the other hand, OSC2 does not show such a difference, which may result in a rather poor performance. Based on a range of FFs for solar cells developed with organic materials reported by different authors, which varies from 0.15 to 0.73 [2,41,42,43], the FF value for OSC1 (0.23) proves that it can be considered as an OSC. In contrast, even when its current density values are better at higher voltages, the resulting FF for OSC2 (0.07) is too low to be considered into the range reported in the literature.

Another interesting parameter to consider is *J_SC_*. Due to its architecture, electrons in OSC1 are collected in the Ag electrode and holes on the ITO side. In OSC2 the process is the opposite, electrons are collected at the ITO and holes in the Ag electrode. It has been demonstrated that this kind of changes in the architecture of solar cells has an effect in the short circuit current density values [2]. Therefore, the difference between the *J_SC_* results obtained for the devices (Table 2) becomes relevant, as it may indicate that in OSC1 one of the recollection processes is more effective than in OSC2. When aminocarbene **3c** was employed as ETL, the CuPc layer became the hole transporting element, and when **3c** acted as HTL, CuPc became the electron transporting layer. As CuPc has been shown to have the required electrical properties to act either as HTL or ETL when in the presence of TTF [44], then the changes in *J_SC_* should be attributed to the role of the Fischer carbene. Having **3c** as the interfacial layer between the active materials and the Ag cathode results in a short circuit current density of 1.037 mA/cm^2^. This is an indicator of an efficient transport of electrons and holes between the respective active and interfacial elements.

The overall results presented above indicate that the aminocarbene with the pyridyl group in position 4, **3c**, is the one that exhibits the best optoelectronic behavior as thin film, which allows it to be used as HTL or ETL. However, its best performance was observed as electron transporting layer. It is interesting to note that the substitution position of the pyridine ring is the only difference among the structures of the three molecules, **3a**–**c**, and this is apparently enough to induce important changes in the optoelectronic properties of films of these complexes and their function in OSCs.

## 3. Experimental

### 3.1. General Information

All the reactions were performed under an inert atmosphere of nitrogen or argon. All reagents were obtained from commercial suppliers and used without further purification. [(Ethoxy)(methyl)methylidene] pentacarbonyl chromium (0), **1,** was prepared from MeLi and Cr(CO)_6_, according to reported procedures [16]. Silica gel (Merck, Kenilworth, NJ, USA, type 60, 0.063–0.200 mm) was used for column chromatography. All compounds were characterized by IR spectroscopy, on a Perkin-Elmer 283B or 1420 spectrophotometer (PerkinElmer, Inc., Waltham, MA, USA), as KBr pellets. Melting points were obtained on a Melt-Temp II apparatus (Barnstead Thermolyne Corporation, Dubuque, IA, USA) and are uncorrected. NMR spectra were recorded on a Bruker Avance III equipment (Bruker Corporation, Billerica, MA, USA) at 300 MHz (^1^H-NMR) and 75 MHz (^13^C-NMR), in acetone-d_6_ and using TMS as reference. The MS (FAB^+^, *m*/*z*) data were obtained on a MStation JMS-700 spectrophotometer (JEOL Ltd., Akishima, Tokyo, Japan). Suitable X-ray quality single crystals of **3b** were grown by slow evaporation of a hexane-dicloromethane mixture at −5 °C. The crystals were mounted on a glass fiber at room temperature, and then placed on a Bruker Smart Apex CCD diffractometer (Bruker Corporation), equipped with Mo Kα radiation; decay was negligible in both cases. Systematic absences and intensity statistics were used in the space group determination. The structure was solved using direct methods. Anisotropic structure refinements were achieved using a full matrix, least-squares technique on all non-hydrogen atoms. All hydrogen atoms were placed in idealized positions, based on hybridization, with isotropic thermal parameters fixed at 1.2 times the value of the attached atom. Structure solutions and refinements were performed using SHELXTL V6.10. 

### 3.2. Synthesis of Pyridyl(aminocarbene)chromium(0) Complexes ***3a**–**c***

[(Ethoxy)(methyl)methylidene] pentacarbonyl chromium (0), **1** (1 g, 3.78 mmol), was dissolved in 10 mL of anhydrous CH_2_Cl_2_, and 3 equiv. of the corresponding (aminomethyl)pyridine, **2**, were added at room temperature. The solution was stirred for 1 h, gradually changing its color from dark red to bright yellow. After this time, the precipitate formed was collected by filtration, washed with cold dichloromethane/hexane and dried in vacuo to give an analytically pure yellow crystalline solid. An extra amount of pure aminocarbene was obtained when the filtrate was reduced to a small volume and chromatographed on a short silica gel column using hexane: acetate 1:1 as eluent.

#### 3.2.1. [(Methyl)(pyridin-2-ylmethanamine)methylidene] Pentacarbonyl Chromium (0), **3a**

Yellow crystalline solid (1.0 g, 82%), m.p. 88–90 °C. ^1^H-NMR (300 MHz, acetone-*d*_6_, ppm): isomer *E* (64.3%) δ 10.90 (s, 1H, N-H), 8.61 (t, *J* = 5.2 Hz, 1H, C-H Py), 7.85 (t, *J* = 7.3 Hz, 1H, C-H Py), 7.42 (d, *J* = 7.8 Hz, 1H, C-H Py), 7.36 (dd, *J* = 6.9, 5.4 Hz, 1H, C-H Py), 4.97 (d, *J* = 4.8 Hz, 2H, -CH_2_-), 2.79 (s, 3H, -CH_3_); isomer *Z* (35.6%) δ 10.67 (s, 1H, N-H), 8.61 (t, *J* = 5.2 Hz, 1H, C-H Py), 7.85 (t, *J* = 7.3 Hz, 1H, C-H Py), 7.52 (d, *J* = 7.3 Hz, 1H, C-H Py), 7.36 (dd, *J* = 6.9, 5.4 Hz, 1H, C-H Py), 5.31 (d, *J* = 4.1 Hz, 2H, -CH_2_-), 2.89 (s, 3H, -CH_3_). ^13^C NMR (75 MHz, acetone-*d*_6_, ppm): isomer A δ 276.4 (C=Cr), 223.4, 218.2 (Cr-CO), 154.5 (C_ipso_), 149.3 (C-H Py), 137.2 (C-H Py), 122.9 (C-H Py), 122.0 (C-H Py), 51.7 (-CH_2_-), 35.9 (-CH_3_); isomer B δ 272.9 (C=Cr), 223.5, 218.2 (Cr-CO), 154.9 (C_ipso_), 149.4 (C-H Py), 137.0 (C-H Py), 123.0 (C-H Py), 122.1 (C-H Py), 57.7 (-CH_2_-), 43.7 (-CH_3_). FT-IR (KBr, cm^−1^): ν 3175 (N-H); 2050, 1893 (Cr-C=O). FT-IR (Thin film, cm^−1^): ν 3177 (N-H); 2055, 1909 (Cr-C=O). MS (FAB^+^, *m*/*z*): 326 [M]^+^_,_ 298 [M^+^-CO], 270 [M^+^-2CO], 242 [M^+^-3CO], 214 [M^+^-4CO], 186 [M^+^-5CO]. HRMS (ESI-TOF): calcd. for C_13_H_10_CrN_2_O_5_ [M]^+^ 325.9995; found 325.9996.

#### 3.2.2. [(Methyl)(pyridin-3-ylmethanamine)methylidene] Pentacarbonyl Chromium (0), **3b**

Yellow crystalline solid (1.12 g, 91%), m.p. 147-148 °C. ^1^H-NMR (300 MHz, acetone-*d*_6_, ppm): δ 11.02 (s, 1H, N-H), 8.63 (s, 1H, C-H Py), 8.56 (br, 1H, C-H Py) 7.81 (br, 1H, C-H Py), 7.42 (br, 1H, C-H Py), 4.98 (s, 2H, -CH_2_-), 2.81 (s, 3H, -CH_3_). ^13^C NMR (75 MHz, acetone-*d*_6_, ppm): δ 277.8 (C=Cr); 223.4, 218.1 (Cr-CO); 149.2 (C_ipso_); 135.1 (C-H Py); 131.7 (C-H Py); 123.7 (C-H Py); 100.0 (C-H Py); 49.0 (-CH_2_-); 35.6 (-CH_3_). FT-IR (KBr, cm^−1^): ν 3175 (N-H); 2050, 1977, 1903 (Cr-C=O). FT-IR (Thin film, cm^−1^): ν 3181 (N-H); 2047, 1975, 1902 (Cr-C=O). MS (FAB^+^, *m*/*z*): 326 [M]^+^_,_ 298 [M^+^-CO], 270 [M^+^-2CO], 242 [M^+^-3CO], 214 [M^+^-4CO], 186 [M^+^-5CO]. HRMS (ESI-TOF): calcd. for C_13_H_10_CrN_2_O_5_ [M]^+^ 325.9995; found 325.9996.

#### 3.2.3. [(Methyl)(pyridin-4-ylmethanamine)methylidene] Pentacarbonyl Chromium (0), **3c**

Yellow crystalline solid (960 mg, 78%), m.p. 134-135 °C. ^1^H-NMR (300 MHz, acetone-*d*_6_, ppm): δ 11.01 (s, 1H, N-H), 8.60 (dd, *J* = 4.5, 1.5 Hz, 2H, C-H Py), 7.34 (d, *J* = 5.9 Hz, 2H, C-H Py), 4.98 (d, *J* = 6.1 Hz, 2H, -CH_2_-), 2.74 (s, 3H, -CH_3_). ^13^C NMR (75 MHz, acetone-*d*_6_, ppm): δ 279.4 (C=Cr); 223.4, 218.0 (Cr-CO); 150.3 (C_ipso_); 144.9 (C-H Py); 122.0 (C-H Py); 50.1 (-CH_2_-); 35.6 (-CH_3_). FT-IR (KBr, cm^−1^): ν 3158 (N-H); 2057, 1902 (Cr-C=O). FT-IR (Thin film, cm^−1^): ν 3161 (N-H); 2051, 1908 (Cr-C=O). MS (FAB^+^, *m*/*z*): 326 [M]^+^_,_ 298 [M^+^-CO], 270 [M^+^-2CO], 242 [M^+^-3CO], 214 [M^+^-4CO], 186 [M^+^-5CO]. HRMS (ESI-TOF): calcd. for C_13_H_10_CrN_2_O_5_ [M^+^] 325.9995; found 325.9996.

### 3.3. Thin Film and Device Manufacture

Three kinds of substrates were used to deposit the aminocarbene complexes films: Corning glass slides, indium tin oxide (ITO) coated glass slides and monocrystalline silicon (100 Si) wafers. They all were subjected to ultrasonic cleaning and degreasing processes in different solvents, prior to use. The deposition process was performed in an evaporation equipment with a tantalum boat. The evaporation rate (4 Å/s), temperature (298 K) and pressure (1 × 10^−6^ Torr) in the vacuum chamber were the same for all the deposition processes. The vacuum in the chamber was achieved by the operation of two pumps: a mechanical pump that generated an initial pressure of 5 × 10^−3^ Torr, and a turbo-molecular pump that allowed us to obtain a 1 × 10^−6^ Torr vacuum in the chamber.

After the characterization of the Fischer aminocarbene films, OSCs were fabricated also by a vacuum deposition process. The materials used for the fabrication of the different OSCs were either purchased from Sigma-Aldrich (Saint Louis, MO, USA) or synthesized as previously indicated in *Materials and synthesis* and purified by temperature-gradient sublimation under high vacuum before use. The solar cells glass/ITO/CuPc/TTF/TCNQ/Carbene/Ag (OSC1) and glass/ITO/CuPc/TCNQ/ TTF/Carbene/Ag (OSC2) were built by sequential sublimation depositions using different evaporation ports. The evaporation rate (1.2 Å/s), temperature (298 K) and pressure (1 × 10^−6^ Torr) in the vacuum chamber were the same for all the OSCs. The ITO coated glass slides were used as transparent bottom contacts, while top contacts were prepared with Ag paint. A schematic representation of both types of cells is shown in Figure 7. In OSC1 TTF was deposited on top of the Fischer aminocarbene, acting as the photosensitive organic semiconductor electron donor layer for the solar cell. After that, TCNQ was deposited, acting as the electron acceptor layer. In OSC2 the positions of the TTF and the TCNQ are inverted (Figure 7). The active area of the devices was 0.0314 cm^2^.

### 3.4. Characterization

During the deposition processes, the films’ thicknesses were monitored with a quartz crystal monitor. The FT-IR analysis was performed for the samples as KBr pellets and films deposited on silicon wafers, on a Nicolet iS5-FT spectrometer (Thermo Fisher Scientific Inc., Waltham, MA, USA). For SEM*,* a ZEISS EVO LS 10 scanning electron microscope (Zeiss International Inc., Göttingen, Germany) operated at a voltage of 20 kV and a focal distance of 25 mm, was used for films on Corning glass substrates. The AFM characterization was performed on a BRUKER INNOVA microscope (Bruker Corporation, Billerica, MA, USA), on films deposited on glass and ITO-coated glass substrates, using the tapping mode. 

The films on glass substrates were submitted to X-ray diffraction analyses, using the θ–2θ technique, on a Rigaku D-max 2100 diffractometer (Rigaku Corporation, Akishima-shi, Tokyo, Japan), with Cu *K*alpha1/*K*alpha2 radiation (1.5406/1.5444 Å). The optical absorption of the films on glass slides was measured on a Unicam spectrophotometer (Thermo Fisher Scientific Inc.), model UV300, in the wavelength range of 200–1100 nm. The current-voltage-luminance characterization was performed using a programmable voltage source, a sensing station with lighting and temperature controller circuits from Next Robotix (Comercializadora K Mox, S.A. de C.V., CDMX, Mexico) and an auto-ranging Keithley 4200-SCS-PK1 pico-ammeter (Tektronix Inc., Beaverton, OR, USA). 

## 4. Conclusions

This work presents an easy and fast method to synthesize a new series of Fischer carbenes with the general formula [Cr(CO)_5_{C(NHCH_2_)Ar}] (Ar = 2-pyridyl (**3a**), 3-pyridyl (**3b**) and 4-pyridyl (**3c**)). The aminocarbene complexes synthetized were characterized and used to prepare thin films. The latter was carried out by a vacuum deposition method that permits a controlled layer-by-layer growth of high purity films. The morphology of the films seems to depend on the molecular structure of the carbene used. The differences observed in the optical and electrical properties of the thin films also seem to be dependent on the substitution position of the pyridyl group in the complex. The optical activation energy values are 4.0, 2.7 and 2.1 eV for amorphous films of aminocarbenes **3a**, **3b** and **3c**, respectively. Aminocarbene **3c** showed the most suitable optoelectronic properties to be used in the manufacture of organic solar cells. Two of such cells, OSC1 and OSC2, were constructed, differing in the position of the semiconductor (TTF and TCNQ) layers used, relative to the aminocarbene film. The incorporation of film **3c** between the Ag electrode and the active layer constituted by films of electron-donor or electron-acceptor semiconductors, favors the contact between them, benefiting the processes of extraction and collection of charges. Fischer aminocarbene **3c** exhibits a better performance in the selective transport of electrons (ETL) and acts as an energy barrier to avoid the migration of holes. Additionally, this film is transparent to the visible radiation of the electromagnetic spectrum and exhibits an optical activation energy and FF within the range of organic materials that can be used in optoelectronic devices.

## Figures and Tables

**Table 1:** Crystal data and structure refinement for **3b**.

Compound	3b
Empirical Formula	C_13_H_10_CrN_2_O_5_
Formula Weight (g mol^−1^)	326.23
Crystal size (nm)	0.471 × 0.323 × 0.21
Color	Yellow
Crystal system	Monoclinic
Space Group	P 2_1_/n
*a* (Å)	7.4665(4)
*b* (Å)	16.6098(8)
*c* (Å)	12.4271(6)
α (°)	90
β (°)	105.505(3)
γ (°)	90
*V* (Å^3^)	1485.08(13)
*Z*	4
D_calc_ (g cm^3^)	1.459
Number of collected reflections	31279
Number of independent reflections (*R*_int_)	4342, *R*_int_ = 0.0479
Maximum and minimum transmission	0.707 and 0.851
Data/restraints/parameters	4342/0/195
Final *R* indices [*l* > 2*σ*(*l*)]	*R* = 0.0369, *wR*2 0.0872
R indices (all data)	*R* = 0.0612, *wR*2 = 0.1008
GoF (*F*^2^)	1.026
Absorption correction method	Multi-scan

**Table 2:** Characteristic parameters of the organic solar cells constructed.

Device	*V_max_* (V)	*J_max_* (mA/cm^2^)	*V_OC_* (V)	*J_SC_* (mA/cm^2^)	FF
OSC1	0.050	0.717	0.150	1.037	0.23
OSC2	0.010	0.121	0.025	0.694	0.07
